# Psychological capital and breakthrough innovation: The role of tacit knowledge sharing and task interdependence

**DOI:** 10.3389/fpsyg.2023.1097936

**Published:** 2023-02-17

**Authors:** Rui Hu, Yingchao Li, Jiayu Huang, Ying Zhang, Rong Jiang, Elizabeth Dunlop

**Affiliations:** ^1^School of International Languages and Cultures, Yunnan University of Finance and Economics, Kunming, Yunnan, China; ^2^School of Logistics and Management Engineering, Yunnan University of Finance and Economics, Kunming, Yunnan, China; ^3^International Business School, Yunnan University of Finance and Economics, Kunming, Yunnan, China; ^4^School of Business, Charles Sturt University, Wagga Wagga, NSW, Australia; ^5^Institute of Intelligence Applications, Yunnan University of Finance and Economics, Kunming, Yunnan, China

**Keywords:** psychology capital, tacit knowledge sharing, task interdependence, breakthrough innovation, positive organizational behavior

## Abstract

Compared with incremental innovation, breakthrough innovation is essential to sustaining competitive advantage, but breakthrough innovation has the characteristics of high standards and strict requirements. As the main body and foundation of enterprises, the attitude and behavior of employees play a vital role in enterprise innovation. Based on the positive organizational behavior theory and knowledge management theory, the purpose of this paper is to investigate the relationship between psychological capital and breakthrough innovation, and we also integrate tacit knowledge sharing and task interdependence into the research framework, so as to further explore the influence mechanism of employees’ psychological capital on breakthrough innovation. Utilizing a quantitative method, this study takes employees of Yunnan coffee enterprises as investigation objects, the data was analyzed using regression analysis through SPSS 24.0, and the existence of mediation was further verified by Bootstrap test. The results showed that the psychological capital of employees have a positive impact on breakthrough innovation; tacit knowledge sharing partially mediates the relationship between psychological capital and breakthrough innovation; and task interdependence plays a moderating role, that is, the stronger the task interdependence, the stronger the influence of employee psychological capital on breakthrough innovation. This study enriches the research on the influencing factors of breakthrough innovation of Yunnan coffee industry, expands the application scenarios of the related theory, emphasizes that the importance of psychological capital and the breakthrough innovation is the result of the interaction and value-added linkage of various internal and external resources.

## 1. Introduction

In May 2020, the Chinese Government formulated and released “S*everal Key Measures for Strengthening Basic Research under the New Situation*,” which was specifically proposed to establish an exemption mechanism for free exploration and disruptive innovation activities and put forward to give tolerance and encouragement to the failure of innovation activities. On August 15, 2022, “*the Action Plan for Improving Enterprise Technological Innovation Capacity (2022–2023)*,” jointly formulated by the Ministry of Science and Technology and the Ministry of Finance of China, was released. The plan clearly puts forward a series of supportive policies and measures, such as preferential tax policies, talent introduction plan, and financial support, to guide various enterprises to increase independent innovation, reduce research and development costs, and improve the internationalization level of enterprise innovation. Based on the guiding ideology of the general direction proposed by the state, the local governments are also constantly implementing a number of favorable innovation policies for enterprises, accelerating the accumulation of innovation elements to enterprises, ensuring that all kinds of enterprises achieve positive results in leading high-quality development through scientific and technological innovation, and helping backbone enterprises to become national strategic scientific and technological forces. Thus, with the increasingly fierce international competition and the turbulence of the market environment, continuous innovation is the only way to maintain effective survival. The prosperity of the country depends on the efforts of all people in society, as every layer of society is interlinked.

Compared with incremental innovation, breakthrough innovation is the key for enterprises to obtain competitive advantages and ensure long-term development (Jansen et al., 2006). Breakthrough innovation is a challenge, which involves the reconstruction and subversion of the existing knowledge, technology and products (Therrien et al., 2011; Wang et al., 2017), and its success will achieve fundamental change. But generally speaking, breakthrough innovation has two characteristics: high yield and high risk. First of all, breakthrough innovation is a subversion of previous mature technologies and markets, so its activities are faced with huge risks and uncertainties (Gilson and Madjar, 2011; Jia, 2018). Secondly, enterprises will get more development opportunities and huge benefits, if the breakthrough innovation can be successful (O'Reilly III and Tushman, 2013). Despite the importance of breakthrough innovation, both theory and practice show that not all companies can profit from innovation, and the failure rate of innovation attempts is still at a high level.

As the main body and foundation of enterprises, the working attitude and ability of the employees determine the work quality of the team, which then affect the achievement of organisational objective degree and the overall performance level of the enterprise. Therefore, most enterprises currently have a relatively reasonable mechanism for employee recruitment, training, incentive and promotion process, so as to train high-quality enterprise successors as far as possible. With the gradual rise of psychology in research fields, the role of psychological capital is also becoming prominent. It refers to an internal positive mental state of an individual (Luthans and Youssef, 2004), usually including four dimensions: self-efficacy, optimism, hope, and resilience. Recent research perspectives on employees’ psychological capital tend to be diversified, covering themes such as personal job satisfaction, job performance, innovative behavior and ability, and so on (Alessandri et al., 2018; Kumar et al., 2022; Okros and Vîrgă, 2022). In general, psychological state will affect the output of individual behavior. As one of the important resources of enterprises, the positive role of psychological capital is far more than we think (Larson and Luthans, 2006).

As the saying goes, “knowledge is power.” In the current society, the knowledge reserve represents the basic abilities of an individual. For both individuals and enterprises, knowledge is the cornerstone of success, the source of wealth growth, and one of the most important factors for gaining a competitive advantage (Du Plessis, 2007). Compared with the easy acquisition, preservation and dissemination of explicit knowledge, tacit knowledge is a kind of more private knowledge that is hidden in the individual’s mind and cannot be compiled with logical thinking. This type of knowledge is difficult to acquire, spread, and preserve directly, and will play a vital role in the development of enterprises (Nonaka and Takeuchi, 1995). Therefore, the identification and acquisition of tacit knowledge is an important way for enterprises to obtain effective information, but the transformation, sharing and application of tacit knowledge is the key for enterprises to achieve breakthrough innovation. Generally speaking, the sharing of tacit knowledge depends on the individual’s subjective willingness, and they will measure the possible pros and cons before deciding whether to share them (Ryan and O’Connor, 2013; Duan et al., 2018). However, we believe that individuals with different levels of psychological capital have different criteria in the measurement of risk and return, which will also affect their willingness to share tacit knowledge. On the other hand, task interdependence can reflect the frequency of communication among team members to some extent (Campion et al., 1996). The stronger the task interdependence, the more necessary communication between members, and the greater the possibility of information or knowledge sharing, which will have a direct or indirect impact on performance.

Yunnan coffee industry has a history of more than 100 years. Although it has unique regional advantages and policy support, the development of Yunnan coffee industry has faced many challenges in the context of the rapid growth of China’s coffee consumption market in recent years. At present, there are more than 420 coffee enterprises in Yunnan Province, but the overall brand effect is small, the deep processing technology is insufficient, the industrial chain is immature, and the innovation ability of some enterprises has been improved slowly. Therefore, Yunnan coffee industry is in urgent need of transformation and upgrading to improve its competitiveness.

Accordingly, this paper aims to explore the relationship and influence mechanism between employee psychological capital, tacit knowledge sharing and enterprise breakthrough innovation by studying the state of Yunnan coffee enterprises. Combined with the role of task interdependence, the purpose is to provide a new perspective for enterprise breakthrough innovation research, and improve the awareness of the importance of employees’ psychological capital. Meanwhile, this research also provides a useful reference for the improvement of team cooperation efficiency and enterprise innovation performance.

## 2. Literature review and hypotheses development

### 2.1. Psychological capital and breakthrough innovation

Psychological capital refers to a positive mental state including self-efficacy, hope, optimism and resilience presented by individuals during their growth and development. All of the four dimensions are the mentality that we need and are necessary to maintain in study and work.

First, self-efficacy is people’s belief in completing a task or work behavior (Bandura, 1977), which can directly affect the individual’s thoughts, motivation, attitudes, and behavior. In another word, it represents a degree of confidence. Confident individuals are often able to master learning methods and essentials quickly, believing that they can use their own professional knowledge and ability to solve different problems and get better outcomes (Abbas and Raja, 2015). It has been shown that employees with high self-efficacy are often more confident in handling complex interpersonal relationships, will actively express and share their views in the team, and build good relationships with their colleagues, thus showing a high level of active socialization (Parker, 1998). Usually, individuals with high self-efficacy are more motivated to choose more difficult tasks (Luthans et al., 2010). Further, self-efficacy can provide psychological support for employees’ creative activities (Malik et al., 2015), and they prefer to choose positive coping strategies against stress to stick to goals (Bandura, 2001; Tang, 2020). As the driving force of individual creativity, self-efficacy has a positive impact on their creative activities, innovative attitudes and behaviors (Gong et al., 2009; Ahlin et al., 2014; Chen et al., 2016; Klaeijsen et al., 2018), including corporate employees and entrepreneurs in different industries.

As the saying goes, no matter how long the night is, the day will come. Hope is an important positive psychological resource, and also a future-oriented emotional variable, that will affect the individual’s ideological attitude and behavior mode of future development (Scioli et al., 2011). When individual development encounters barriers and faces great stress, hope as a positive cognitive model can help them to actively meet difficulties and challenges (Snyder and Lopez, 2002; Valle et al., 2006). Employees with high levels of hope are better at setting challenging goals and seeking all available resources to accomplish them. Even when they encounter difficulties, they can always keep a positive attitude and constantly break through themself to produce more innovative behaviors (Luthans et al., 2008). Most studies agree with the positive role of hope, including in physical and mental health, job satisfaction, job motivation, and job performance (Peterson and Luthans, 2003; Lee and Na, 2013; Fourati and Attitalah, 2018).

In addition, optimism, as a typical cognitive feature, was studied by most early scholars in combination with physical and mental health. Over time, some hidden characteristics have also been gradually introduced into social science research. Optimism is generally defined as the expectation of positive future outcomes, and there is no doubt that optimism has a positive impact on human health (Scheier and Carver, 1985; Friedman et al., 1992). Optimism encourages individuals to remain enthusiastic about their life and work (Hmieleski and Baron, 2009), thus becoming more likely to set challenging goals, and pursue innovative activities to seize market opportunities for higher performance (Fang et al., 2012). What is important is that they can withstand failures and setbacks, not fall into depression and anxiety, and get out of trouble quickly (Gibbons et al., 2000). Shepperd et al. (2017) believe that optimism plays a role in improving interpersonal relationships, and some scholars also believe that optimistic managers will have an impact on their strategic choices and the development and performance of enterprises (Dushnitsky, 2010; Papenhausen, 2010; Paolillo et al., 2015), but they should not be blindly optimistic.

What’s more, resilience is one of the most important factors affecting individual development and represents an adaptive behavior ability (Luthans et al., 2008; Näswall et al., 2019). That is, individuals can recover in the face of difficulties in life, work failure and other problems quickly, and have the courage to start all over again (Bardoel et al., 2014; Britt et al., 2016; Linnenluecke, 2017), so as to achieve better growth and development. Studies have shown that resilience can improve employee job satisfaction and happiness (Luthans et al., 2007; Kuntz et al., 2017), develop good social skills, maintain a good organizational atmosphere (Cooke et al., 2019), and thus improve performance.

Although the success of the enterprise breakthrough innovation can generate huge benefits, before the success the breakthrough innovation activities will face huge risks, because it is the subversion of the previous mature technologies and markets. Therefore, as the main body of the enterprise, in this case, in addition to excellent skills, the positive psychological state of employees is a stabilizer for the enterprise. As mentioned above, positive psychological capital enables employees and teams in a better working condition, realize positive organizational behavior, remain optimistic about innovation activities, recover quickly from setbacks and failures, and then have the confidence and courage to try again to achieve final success. Fang et al. (2019) mentioned in his research that psychological capital, as a positive mental state, can enhance employees’ motivation to innovate. Gao et al. (2020) takes entrepreneurs as research objects and concludes that entrepreneurs with a higher level of psychological capital have stronger innovation initiative and are more likely to generate creative innovation behaviors. Kumar et al., (2022), based on the study of Indian hotel industry employees, believes that psychological capital has a positive correlation with its innovative work behavior. Some scholars also believe that entrepreneurs who are usually full of optimism and hope are more capable of helping enterprises to innovate their business models (Fourati and Attitalah, 2018; Zhou et al., 2022). Dórdio et al. (2022) studied the relationship between team psychological capital and innovation by taking team learning as the mediating variable, and the results showed that the higher the level of the team psychological capital, the more the innovation output. Yuan and Chai (2020) investigated high-tech enterprises and concluded that employees with higher innovation ability generally showed a higher level of psychological capital. Le (2020) suggested that enriching employees’ psychological capital may be a favorable choice and an important method to improve enterprises’ innovation ability. Alshebami (2021) focused on small and medium-sized enterprises in Saudi Arabia, arguing that psychological capital can improve employee job satisfaction and motivate them to try innovation. Previous studies have confirmed that psychological capital is closely related to innovation performance (Judge and Bono, 2001; Tang, 2020; Brunetto et al., 2022). Thus, it is hypothesized that:

*Hypothesis 1*: Psychology capital is positively related to Breakthrough Innovation.

### 2.2. The mediating role of tacit knowledge sharing

#### 2.2.1. Psychology capital and tacit knowledge sharing

Knowledge-sharing behavior is a communication process between knowledge providers and knowledge seekers, and the purpose of communication is to obtain the required information and internalize the knowledge (Wang and Noe, 2010; Vuori and Okkonen, 2012). As most scholars agree, knowledge is usually divided into explicit knowledge and tacit knowledge (Nonaka and Takeuchi, 1995), and the economic value of the two is different; with tacit knowledge being more valuable (Reychav and Weisberg, 2010). Holste and Fields (2010) have proposed that tacit knowledge is the most core strategic resource of an enterprise in the era of knowledge economy. Therefore, it can be seen that promoting the sharing of tacit knowledge and playing the role of tacit knowledge will help the organization to break through new technologies, gain continuous competitive advantages, and achieve sustainable development.

Tacit knowledge is a kind of knowledge hidden in the individual’s mind and unique to the individual, including their own learning methods, working skills, inspiration, etc. Therefore, the sharing of tacit knowledge is not an obligation of an individual, they have the right to choose whether to share it or not (Jones and Jordan, 1998). On the other hand, as employees are often faced with fierce competition, they are wary of sharing their hidden knowledge, because they worry about losing their unique value and competitive advantage (George, 1995; Taegoo and Gyehee, 2013). Therefore, how to enhance employees’ willingness to share tacit knowledge has become an important topic (Terhorst et al., 2018). Wang and Noe (2010) have suggested that there is a range of factors affecting knowledge sharing, including the organizational level, team level, and individual level. Employees’ willingness to communicate, cooperate and share knowledge is highly related to their level of psychological capital (Ghazinour et al., 2014).

Previous studies have explored the relationship with knowledge sharing through the dimension of psychological capital, and most of them recognize the positive role of psychological capital (Kollock, 1999; Quigley et al., 2007; Shin et al., 2007; Hau and Kim, 2011; Panahi et al., 2016). Thus, it is hypothesized that:

*Hypothesis 2*: Psychological capital is positively related to tacit knowledge sharing.

#### 2.2.2. Tacit knowledge sharing and breakthrough innovation

Enterprises usually treat breakthrough innovation with high standards and strict requirements. So almost every major accomplishment in enterprises with the numerous exploration and attempts. We believe that the positive mental state of employees can promote the sharing of tacit knowledge, and the tacit knowledge sharing with high economic value can help to maintain a good communication relationship among team members to better cooperate and achieve breakthroughs.

Although there is little direct research on tacit knowledge sharing and breakthrough innovation, we can interpret the relationship between them from different perspectives. As the basis of individual innovation behavior, the generation of innovative thinking is the result of knowledge exchange, accumulation and application (Oliveira et al., 2015). Studies have shown that knowledge sharing is part of learning within an organization, the information and knowledge sharing among members will contribute greatly to the team and the organization (Wang and Wang, 2012; Kim and Lee, 2013), which can help the employees develop creative thinking and innovation behavior (Baradarani and Kilic, 2018; Gao et al., 2020; Kumar et al., 2022), improve market acuity and conduct innovative activities in a timely and effective manner (Lin C. P., 2007; Lin H. F., 2007). García-Álvarez (2015) concluded that tacit knowledge can positively promote innovation in products and processes. Jiang and Chen (2018) have confirmed that team members actively share their knowledge and skills, which will help promote the team’s knowledge integration ability, improve the internal knowledge integration mechanisms and thus ensure the smooth progress of innovation activities. Tacit knowledge can break through, enrich and expand the existing internal knowledge structure and database, which is more conducive to the implementation of breakthrough innovation (Mascitelli, 2000; Prabhu et al., 2005; Laursen and Salter, 2006). In general, both tacit knowledge and knowledge sharing will have a significant impact on enterprise innovation. Thus, it is hypothesized that:

*Hypothesis 3*: Tacit knowledge sharing is positively related to breakthrough innovation.

#### 2.2.3. Psychology capital, tacit knowledge sharing and breakthrough innovation

Based on the above analysis, we can see that there is a certain correlation between employee psychological capital, tacit knowledge sharing and breakthrough innovation. First of all, we know that employees with high psychological capital have rich knowledge reserve, believe in their own abilities, tend to choose challenging and innovative tasks, and achieve innovative performance by constantly breaking through themselves (Zhou et al., 2022). Compared with employees with lower levels of psychological capital, they are more able to accumulate experience, gain experience from failure, and learn new knowledge. On the other hand, since tacit knowledge sharing is not an employee’s job duty and obligation, whether employees are willing to share their unique knowledge and skills depends on their personal thoughts and psychological factors to some extent (Ghazinour et al., 2014). If an employee has a high level of psychological capital, it means that he often keeps himself in a positive mood and working state, which will strengthen his collectivism concept and thus produce the willingness to share tacit knowledge (Chiu et al., 2017). However, employees with low level of psychological capital usually have strong exclusivity and tend to be individualistic, mainly due to lack of trust in colleagues, so that they are less willing to share knowledge (Mura et al., 2021), especially tacit knowledge. Alves and Pinheiro (2022) also proved that individual psychological factors will affect the sharing of tacit knowledge within a team. Secondly, with the gradual transformation of economy, knowledge is increasingly recognized as an intangible and valuable resource (Thomas and Gupta, 2022). Explicit knowledge that is readily available to the general public lacks competitiveness in its exploitable value, but the sharing of tacit knowledge is an effective and important way to obtain unique information (Noori Sepehr and Keikavoosi-Arani, 2019). For enterprises, although it is difficult to acquire tacit knowledge, it has high economic value and can help enterprises make breakthrough progress.

In general, because tacit knowledge is private, its sharing almost depends on the will of the individual. Therefore, psychological capital will affect individuals’ sharing of tacit knowledge, and employees with different levels of psychological capital will have different willingness to share tacit knowledge. The sharing of tacit knowledge can transform individual knowledge into organizational knowledge, improve work efficiency and develop innovative behaviors (Gao et al., 2020), thus affecting organizational innovation efficiency and the possibility of radical innovation (Mascitelli, 2000). Therefore, according to the principle of mediating variable selection, tacit knowledge sharing has a strong correlation with psychological capital and breakthrough innovation, and this study believes that tacit knowledge sharing will play a mediating role. Thus, it is hypothesized that:

*Hypothesis 4*: Tacit knowledge sharing mediates the relationship between psychological capital and breakthrough innovation.

### 2.3. The moderating role of task interdependence

Generally speaking, task interdependence reflects the extent of how interdependent team members depend on each other while completing their work, including the required knowledge, information, materials, and a series of behaviors (Wageman, 1995; Wageman and Gordon, 2005; Yang, 2020). The degree of task interdependence determines the frequency of communication between team members and the efficiency of teamwork. Highly interdependent work requires members to strengthen communication and coordination, allocate tasks reasonably, and make full use of their knowledge and skills to achieve their work goals together (Wageman and Gordon, 2005; Han and Bai, 2014; Fong et al., 2018). On the other hand, when the members realize that they need the help of other members to complete their work, they will automatically manage their emotions, reduce the generation of speculation, take the initiative to exchange and share information with the members, and improve their knowledge reserve (Van Der Vegt et al., 2000; Vidyarthi et al., 2014). The interdependence of tasks facilitates employees to produce positive organizational civic behavior (Bachrach et al., 2006), enhance members’ sense of collective responsibility and honor, weaken knowledge hiding (Černe et al., 2014), improve internal creativity (Gilson and Shalley, 2004), and promote the achievement of common goals. In contrast, with low task interdependence, members can perform their work independently, without relying on the help of other members, and in turn have less willingness to share their own knowledge and information.

Employees with a high level of psychological capital can just meet the requirements of task interdependence, that is, active communication and cooperation. They are good at communication, can maintain a good communication relationship, and are willing to share their knowledge and information, so as to help improve the teamwork ability and work efficiency. Although there are few direct studies on task interdependence, psychological capital and breakthrough innovation performance, task interdependence as a moderator variable has been relatively mature. Some scholars have demonstrated the moderating role of task interdependence between knowledge management and creativity (Staples and Webster, 2008; Hon and Chan, 2013). Fong et al. (2018) have proposed that the stronger the task interdependence, the weaker the negative relationship between knowledge hiding and team creativity. Through previous studies, it can be found that task interdependence can moderate the individual’s behavior, psychology, and team performance. Thus, it is hypothesized that:

*Hypothesis 5*: Task interdependence moderates the relationship between psychological capital and breakthrough innovation.

**Figure 1:**
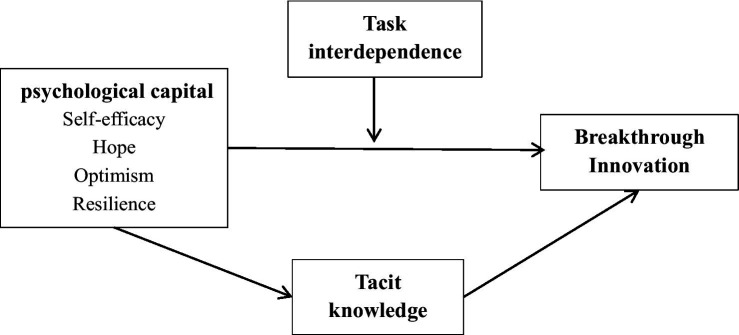
Research framework.

Figure 1 presents our research framework.

## 3. Methodology

### 3.1. Data collection

First, as a pilot study, 20 academics from universities in China were invited to evaluate the validity and reliability of the proposed questionnaire. Secondly, with the assistance of the Chinese Chamber of Commerce List, we randomly selected 15 key enterprises in the coffee industry in Yunnan Province, covering Kunming, Baoshan and Pu'er cities. We distributed the questionnaires to the employees of these enterprises. Due to the spread of the COVID-19 pandemic, the data was gathered *via* the standardized online platform. The survey period was 6 weeks. Finally, a total of 500 responses were collected, with 24 cases contained over 25% missing value and 56 cases failed to satisfy the criteria for the research, resulting in a final data set of 420 usable questionnaires, and the effective response rate of the questionnaire was 84%.

Our questionnaire includes five parts: basic information of individuals and enterprises, psychological capital of employees, tacit knowledge sharing, task interdependence and breakthrough innovation. The questionnaire of each variable adopts the mature scale at home and abroad, and has been revised under the guidance of experts. The questionnaire was mainly filled out by employees of coffee enterprises in Yunnan province, who answered relevant questions according to their own actual conditions to assist us in collecting their psychological capital information. Among the respondents, 56.4% are male and 43.6% female; 56.9% are employees under 40, 20.7% are 41–50, 22.3% are over 50; 52.6% had college degree or below, 36.4% had bachelor’s degree, 11% had master’s degree or above; In terms of working years, 30.5% for 2–5 years, 28.8% for 6–10 years, and 28.6% are over 10 years. Specific information can be given in Table 1.

**Table 1:** Sample characteristics.

Variables	Classification	*N*	Percent
Gender	Male	237	56.4
	Female	183	43.6
Age	18–25	32	7.6
	26–30	79	18.8
	31–40	128	30.5
	41–50	87	20.7
	51–60	72	17.1
	>60	22	5.2
Educational background	Technical secondary school and below	107	25.5
	Junior college	114	27.1
	Bachelor’s degree	153	36.4
	Master’s degree	41	9.8
	PhD degree and above	5	1.2
Working life	<1 year	51	12.1
	2-5 years	128	30.5
	6-10 years	121	28.8
	10 years and above	120	28.6
Type of job	General staff	290	69.0
	Low-level managers	75	17.9
	Middle managers	45	10.7
	Senior managers	10	2.4
Enterprise size	1–99 people	116	27.6
	100–499 people	108	25.7
	500–1,999 people	114	27.1
	2,000–4,999 people	60	14.3
	>5,000 people	22	5.2
Team size	<5 people	31	7.38
	6–10 people	72	17.14
	11–15 people	152	36.2
	15–20 people	85	20.24
	>20 people	80	19.05
Type of enterprise	State-owned enterprise	82	19.52
	Private enterprise	176	41.91
	Foreign-owned enterprise	91	21.67
	Public institution	55	13.10
	Other types	16	3.81
Team life	<1 month	36	8.571
	1–6 months	82	19.524
	6–12 months	88	20.952
	1–2 years	90	21.429
	>2 years	124	29.524

### 3.2. Measurements of variables

#### 3.2.1. Psychological capital

Psychological capital was measured with the scale which was designed by Luthans et al. (2007). The scale contains four dimensions: self-efficacy, hope, resilience, and optimism, each with 6 items and a total of 24 items. All items were scored on a five-point Likert-type scale (1 = strongly disagree, 5 = strongly agree). Some sample items are: “I can think of many ways to reach my current goals”; “I am confident that I could deal efficiently with unexpected events.” The Cronbach’s alpha coefficient of the overall scale was 0.972, and the KMO value of the scale was 0.970 with significant Bartlett test results, and the cumulative variance contribution rate was 65.136%. Factor loading coefficient is between 0.715 and 0.803.

#### 3.2.2. Breakthrough innovation

For the measurement of breakthrough innovation, we mainly draw on the scales developed by Zhou and Li (2012) and Alexander and Van Knippenberg (2014). The scale includes four items, and some sample items are: “The company has introduced brand-new technologies and ideas for innovation”; “The company has developed brand-new products.” These items are rated on a five-point Likert scale from 1 for “Strongly Disagree” to 5 for“Strongly Agree.” The Cronbach’s alpha coefficient of the overall scale was 0.888, and the KMO value of the scale was 0.826 with significant Bartlett test results, and the cumulative variance contribution rate was 75.018%. Factor loading coefficient is between 0.711 and 0.742.

#### 3.2.3. Tacit knowledge sharing

Tacit knowledge sharing was measured by 6 items scale developed by Scott and Bruce (1994). Examples of items in this section are: “I would like to share my work experience with my colleagues”; “I am willing to share my unique expertise if my colleagues need and request”; “I will share my ideas and inspiration with my colleagues.” The Cronbach’s alpha coefficient of the overall scale was 0.924, and the KMO value of the scale was 0.911 with significant Bartlett test results, and the cumulative variance contribution rate was 72.483%. Factor loading coefficient is between 0.7 and 0.826.

#### 3.2.4. Task interdependence

For the measurement of breakthrough innovation, we utilized the scale developed by Van Der Vegt (2000). This scale has five items, including “I need information and opinions from my colleagues to do my job well”; “Team members need to cooperate to do the job well”; “Team members need to communicate regularly on work-related issues.” The Cronbach’s alpha coefficient of the overall scale was 0.884, and the KMO value of the scale was 0.871 with significant Bartlett test results, and the cumulative variance contribution rate was 68.85%. Factor loading coefficient is between 0.651 and 0.867.

The KMO value of the overall questionnaire was 0.968 if the Bartlett-test result was significant, which was suitable for the next analysis.

## 4. Results

### 4.1. Descriptive statistics and correlation analysis

Table 2 shows each variable’s mean and standard deviation as well as the correlation of the variables. From the results shown in the table, we can find that all the variables are significantly positively correlated. This means there is a significant positive correlation between psychological capital, tacit knowledge sharing and breakthrough innovation.

**Table 2:** Descriptive statistics and correlation analysis.

Variables	Mean	SD	Psychology capital	Tacit knowledge sharing	Breakthrough innovation	Task interdependence
Psychology capital	3.0586	0.91654	1			
Tacit knowledge sharing	3.0397	0.91493	0.606^**^	1		
Breakthrough innovation	3.1074	0.85145	0.515^**^	0.463^**^	1	
Task interdependence	3.2907	0.95642	0.329^**^	0.308^**^	0.578^**^	1

^**^Correlation is significant at the 0.01 level (two-tailed).

### 4.2. Assessment of reliability and validity

The reliability and validity of the measurement model were examined using Cronbach’s alpha and Confirmatory Factor Analysis (CFA). First, the Cronbach’s alpha coefficient of each scale ranged from 0.884 to 0.972, while construct reliability (CR) values were greater than 0.7 and ranged from 0.889 to 0.972, it exhibited internal consistency and the scale had good reliability for analysis. The Average Variance Extracted (AVE) values in this study ranged between 0.621 and 0.67. This is greater than the prescribed value of 0.50, which is indicated the convergent validity. The specific results are shown in Table 3. In addition, if the square root of the AVE of a construct is greater than the value of its inter-correlations with other constructs, then it has an excellent discriminative validity. The results are shown in Table 4, and the values on the slash are greater than the others. The results of Harman’s single factor test showed that the variation indicated by the single factor solution remained below the required level of 40%.

**Table 3:** Validity test results.

Variable	Item	Factor loading	Cronbach’s alpha	AVE	CR
Psychology capital	PC1	0.744	0.972	0.635	0.972
	PC2	0.735			
	PC3	0.769			
	PC4	0.741			
	PC5	0.74			
	PC6	0.777			
	PC7	0.765			
	PC8	0.737			
	PC9	0.761			
	PC10	0.762			
	PC11	0.715			
	PC12	0.758			
	PC13	0.756			
	PC14	0.751			
	PC15	0.803			
	PC16	0.748			
	PC17	0.774			
	PC18	0.759			
	PC19	0.77			
	PC20	0.786			
	PC21	0.794			
	PC22	0.8			
	PC23	0.77			
	PC24	0.752			
Breakthrough innovation	BI1	0.712	0.888	0.67	0.89
	BI2	0.711			
	BI3	0.742			
	BI4	0.713			
Tacit knowledge sharing	KS1	0.753	0.924	0.669	0.924
	KS2	0.786			
	KS3	0.826			
	KS4	0.817			
	KS5	0.783			
	KS6	0.700			
Task interdependence	TI1	0.776	0.884	0.621	0.889
	TI2	0.651			
	TI3	0.867			
	TI4	0.865			
	TI5	0.766			

AVE > 0.5 or CR > 0.7, indicating a high polymerization validity.

**Table 4:** Results of Pearson correlation analysis between factors and AVE square root values.

	Psychology capital	Breakthrough innovation	Tacit knowledge sharing	Task interdependence
Psychology capital	0.797			
Breakthrough innovation	0.516	0.819		
Tacit knowledge sharing	0.603	0.463	0.818	
Task interdependence	0.329	0.578	0.308	0.788

The number on the diagonal is the root value of the AVE for this factor.

We also evaluate the fit of the model by some goodness of fit indices, such as root mean square error of approximation (RMSEA), comparative fit index (CFI), root mean square residual (RMR), and nonnormed fit index (NNFI). The indices of the final model fit reported that overall fit was within range of acceptance with *χ*^2^/df = 2.902 < 3, RMSEA = 0.067, RMR = 0.048, CFI = 0.915, NNFI = 0.909. Table 5 shows the specific indicators and judgment criteria. Since the result of the model fitting will be affected by many factors not all of the indicators will achieve very good evaluation results. Usually, we only consider whether most of the indicators meet the evaluation criteria.

**Table 5:** Fit indices.

Indicators	Judgment	Score
X^2^/df	<3	2.902
RMSEA	<0.10	0.067
RMR	<0.05	0.048
CFI	>0.9	0.915
NNFI	>0.9	0.909

df, Degree of freedom; X^2^, Chi-Square; RMSEA, Root mean square error of approximation; CFI, Comparative fit index; RMR, Root mean square residual; NNFI, Nonnormed fit index.

### 4.3. Hypotheses testing

#### 4.3.1. Tests of the relationship between psychological capital and breakthrough innovation

Most of the regression analysis in this article was performed using SPSS 24.0 software. Hierarchical regression was used to examine the relationship between psychological capital and breakthrough innovation. We constructed regression Model 1 and Model 2 with breakthrough innovation as the dependent variable, as shown in Table 6. In Model 1, some control variables were introduced: gender, age, educational background, working life, enterprise size, type of job and enterprise and so on. Model 2 added independent variable psychological capital on the basis of Model 1. We measure the multicollinearity of the independent variables by testing the variance inflation factor (VIF), and it was found that the maximum VIF was less than 5, and the Durbin-Watson value were also within reasonable limits, indicating that there was no serious multicollinearity problem in this study. After the addition of psychological capital, the *F* value of Model 2 increased to 14.864 and was significant, *R*^2^ also increased to 0.286 and the coefficient of PC is 0.485, indicating that psychological capital has a significant positive impact on breakthrough innovation (*t* = 11.918, *p* < 0.001), and Hypotheses 1 was verified.

**Table 6:** Hierarchical regression analysis of Psychological Capital and Breakthrough Innovation.

Model 1				Model 2			
Variable	Regression coefficient	*t*	*p*		Regression coefficient	*t*	*p*
Const	3.816***	7.963	0.000	Const	2.072***	4.728	0.000
PC				PC	0.485***	11.918	0.000
Gender	−0.324**	−2.206	0.028	Gender	−0.13	−1.016	0.310
Age	−0.041	−0.586	0.558	Age	0.001	0.008	0.993
Educational background	0.017	0.373	0.709	Educational background	0.001	0.035	0.972
Working life	−0.058	−0.601	0.548	Working life	−0.056	−0.67	0.503
Enterprise size	−0.046	−1.192	0.234	Enterprise size	−0.076**	−2.285	0.023
Type of job	0.054*	1.657	0.098	Type of job	0.004	0.068	0.946
Team size	−0.018	−0.469	0.639	Team size	−0.054*	−1.657	0.098
Type of enterprise	0.048	1.131	0.259	Type of enterprise	0.066*	1.788	0.075
Team life	0.061**	2.122	0.034	Team life	0.031	0.848	0.397
*R* ^2^	0.038			*R* ^2^	0.286		
Adjusted *R*^2^	0.014			AdjustedR^2^	0.267		
Δ*R*^2^	0.038			ΔR^2^	0.249		
*F*	*F* = 1.595, *p* = 0.105			*F*	*F* = 14.864, *p* = 0.000***		

***p < 0.001, **p < 0.01, *p < 0.05; PC, psychological capital. Model 1 is the model of control variables.

#### 4.3.2. Tests of the mediating effect of tacit knowledge sharing

According to the mediation effect testing step proposed by Baron and Kenny (1986), we first used stepwise regression to test the mediating effect of tacit knowledge sharing in the relationship between psychological capital and breakthrough innovation. First, Model 3 in Table 7 has shown that the coefficient of PC is 0.586 and the effect was significant (*p* < 0.001), which is indicated that psychological capital was positively related to tacit knowledge sharing, Hypotheses 2 was verified. Second, from Model 4 we can see that the regression coefficient of TKS was 0.428 and the effect was significant (*p* < 0.001), this regression result has proved that tacit knowledge sharing can have a positive impact on breakthrough innovation, Hypotheses 3 was verified. Third, Model 5 added the independent variable psychological capital and mediator variable tacit knowledge sharing. The results showed that both coefficients remained significant, they were 0.353 (*p* < 0.001) and 0.225 (*p* < 0.001), proving that tacit knowledge sharing plays a mediating role in the relationship between psychological capital and breakthrough innovation. The above model tests are all based on the results validated by Model 2, that is, the positive impact of psychological capital on breakthrough innovation. Table 7 shows the more details.

**Table 7:** Regression analysis of the mediating effect of Tacit knowledge sharing.

	Model 3			Model 4			Model 5		
Variables	Regression coefficient	*t*	*p*	Regression coefficient	*t*	*p*	Regression coefficient	*t*	*p*
Const	1.893***	4.321	0.000	2.102***	4.579	0.000	1.646***	3.764	0.000
PC	0.586***	14.403	0.000				0.353***	7.24	0.000
TKS				0.428***	10.264	0.000	0.225***	4.664	0.000
Gender	0.193**	−2.183	0.030	−0.006	−0.066	0.948	0.017	0.199	0.843
Age	0.009	−0.145	0.885	−0.016	−0.252	0.801	0.002	0.042	0.967
Educational background	0.03	−0.74	0.460	0.022	0.525	0.600	0.008	0.206	0.837
Working life	0.078	−0.932	0.352	−0.024	−0.274	0.784	−0.038	0.471	0.638
Enterprise size	0.019	0.573	0.567	−0.07**	−2.023	0.044	−0.081**	2.474	0.014
Type of job	0.034	−0.601	0.548	0.041	0.714	0.475	0.011	0.208	0.835
Team size	0.01	0.278	0.781	0.053	1.39	0.165	0.063*	1.768	0.078
Type of enterprise	0.027	−0.835	0.404	−0.025	−0.738	0.461	−0.048	1.505	0.133
Team life	0.052	1.396	0.164	0.019	0.498	0.619	0.02	0.546	0.585
*R* ^2^	0.382			0.235			0.322		
Adjusted *R*^2^	0.364			0.214			0.301		
*F*	*F* = 22.926, *p* = 0.000***			*F* = 11.397, *p* = 0.000***			*F* = 16.132, *p* = 0.000***		
Bootstrap test				Mediating effect	SE		95% Boot CI	Result	
Psychological capital Tacit knowledge sharing breakthrough innovation				0.132	0.036		[0.068–0.216]	Partial mediation	

***p < 0.001, **p < 0.01, *p < 0.05. PC, psychological capital; TKS, Tacit knowledge sharing; BI, Breakthrough Innovation; TI, Task interdependence.

In addition, to further verify the mediating effect of tacit knowledge sharing, we used the Bootstrap sampling test. The results are also shown in Table 7. The confidence level was set to be 95% and the number of random sampling was set to be 1,000. It is generally believed that if the 95% confidence interval of the distribution does not contain 0, the mediating effect is significant. It can be seen that the lower limit is 0.068 and the upper limit is 0.216, excluding 0, indicating that the tacit knowledge sharing plays a partial mediating role. Thus, Hypotheses 4 was verified.

#### 4.3.3. Tests of the moderating effect of task interdependence

**Figure 2:**
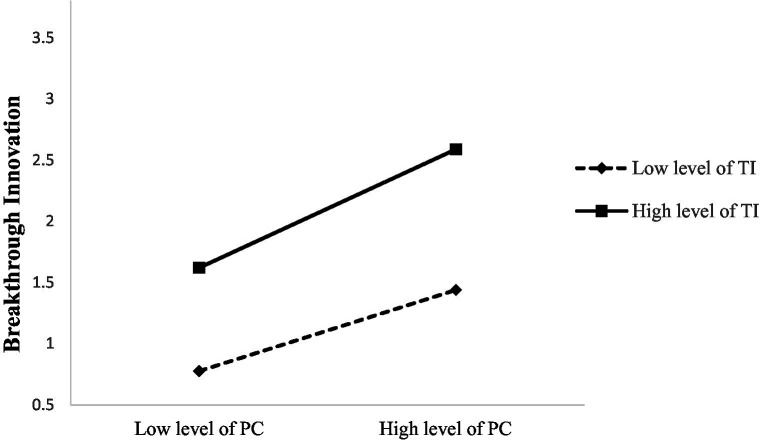
The moderating effect of task interdependence. PC, psychological capital; TKS, Tacit knowledge sharing; TI, Task interdependence.

To verify the moderating effect of task interdependence, we standardized the variables to generate interactive item about psychology capital and task interdependence. We found that in Table 8, the coefficient of “PC*TI” which is an interaction term, is 0.163 and significant (*p* < 0.001). This suggests that task interdependence can effectively adjust the relationship between psychological capital and breakthrough innovation. Thus, Hypotheses 5 were supported. Meanwhile, Figure 2 is designed to more intuitively show the moderating effect of task interdependence. As shown in the figure, the stronger the task interdependence, the more likely employees are to improve their psychological capital level and then play a positive role in breakthrough innovation.

**Table 8:** Regression analysis of the moderating effect of task interdependence.

Model 6				
Variables	Regression coefficient	SE	*t*	*p*
Const	2.701***	0.505	5.345	0.000
PC	0.198	0.123	1.607	0.109
TI	0.066	0.107	0.611	0.541
PC*TI	0.163***	0.035	4.631	0.000
Gender	0.026	0.075	0.343	0.732
Age	0.005	0.051	0.096	0.924
Educational background	0.012	0.034	0.356	0.722
Working life	0.049	0.07	0.695	0.487
Enterprise size	0.059**	0.028	2.082	0.038
Type of job	0.006	0.047	0.136	0.892
Team size	0.048*	0.028	1.751	0.081
Type of enterprise	0.062**	0.031	2.011	0.045
Team life	0.003	0.031	0.081	0.935
*R* ^2^	0.496			
Adjusted *R*^2^	0.479			
Δ*R*^2^	0.496			
*F*	*F*(13, 406) = 30.679, *p* = 0.000***			

***p < 0.001, **p < 0.01, *p < 0.05. PC, psychological capital; TKS, Tacit knowledge sharing; BI, Breakthrough Innovation; TI, Task interdependence.

## 5. Conclusion and discussion

### 5.1. Conclusion

With the development of the society, the current scholars pay more attention to the psychological capital of employees. This study selected the Yunnan coffee industry as the research object to explore whether the psychological capital of employees will have an impact on enterprise breakthrough innovation, and consider the role of tacit knowledge sharing and task interdependence, to further explore the influence mechanism. Based on the analysis results, we have the following conclusions:

First, psychological capital has a significant and positive impact on enterprise breakthrough innovation. Psychological capital represents a positive attitude; the higher the psychological capital level of employees, the more inclined to choose challenging and innovative tasks, use perseverance to accomplish goals, not afraid of difficulties and setbacks, with resilience, can quickly recover from failure and sum up experience, provide a stable foundation and good resources for enterprises to achieve breakthrough innovation.

Second, tacit knowledge acquisition plays a partially mediating role in the relationship between psychological capital and breakthrough innovation performance. For an enterprise, knowledge is the foundation of achievement. If the employees lack the rich knowledge reserve, then the enterprise lacks the core and cannot make any innovation attempt, especially the tacit knowledge. Our study concluded that employees with a higher level of psychological capital have a stronger willingness to share tacit knowledge. Sharing tacit knowledge among team members is conducive to brainstorming activities, generating more innovative ideas and behaviors, and thus improving innovation performance. The tacit knowledge has high economic value and can help enterprises to gain more competitive advantages.

Third, task interdependence plays a moderating role in the relationship between psychological capital and breakthrough innovation performance. Task interdependence can influence the frequency and quality of communication among members, reduce knowledge hiding, promote knowledge sharing, and thus improve team creativity and innovation performance. In a word, with the enhancement of task interdependence, the role of psychological capital in promoting breakthrough innovation of the enterprise will increase.

### 5.2. Theoretical contribution and practical implications

#### 5.2.1. Theoretical contribution

The study makes a variety of theoretical contributions to the literature. First of all, in the past, many scholars have studied the impact of employees’ psychological factors on their own behavior or performance, but this study is the first to combine psychological capital, tacit knowledge sharing and enterprise breakthrough innovation. No studies have attempted to link the current model to the development of the coffee industry in Yunnan Province. This research has enriched the research content and expanded the research scope by analyzing it across different levels.

Secondly, many existing literatures focus on the impact of employees’ psychological capital on their own ability and work performance, and there are insufficient studies on how psychological capital promotes performance at the enterprise level. The empirical results of this study show that the high level of psychological capital of employees will improve the possibility of enterprises to achieve breakthrough innovation. The cross-level analysis complements the relevant research in this field.

Finally, based on positive organizational behavior theory, social exchange theory, job demand-resource model and knowledge management theory, this paper discusses the impact of employee psychological capital and tacit knowledge sharing on enterprise breakthrough innovation. At the same time, we also consider the moderating effect of task interdependence. This study extends the application range of these theories and enriches the research achievements in related fields.

#### 5.2.2. Practical implications

Our results have practical implications for coffee enterprises in Yunnan Province. First of all, employees’ psychological capital is conducive to enterprises’ breakthrough innovation. Therefore, it is beneficial for enterprises to pay attention to the psychological capital of candidates in the recruitment process. As a kind of positive psychological resources, psychological capital can be developed through training and other ways. Enterprises should pay attention to the positive role of employees’ psychological capital, provide strong organizational support and complete working conditions, enhance employees’ sense of organizational integration and master awareness, improve the level of employees’ psychological capital through effective ways, and then make beneficial contributions to the organization.

Secondly, our research also shows that psychological capital can play a role in breakthrough innovation through tacit knowledge sharing. Therefore, it is very important for enterprises to pay attention to tacit knowledge sharing. The coffee industry in Yunnan province is faced with the problems of not getting the industry trends in time and not being well-informed, so it needs to expand the source channels constantly. Within the enterprise, knowledge sharing among employees is an effective way to obtain resource information. Tacit knowledge sharing among members will not only get the knowledge needed within the organization, but can also be a way to obtain potential external information and resources. Enterprises should give incentives to encourage and recognize the knowledge-sharing behavior of employees, and try to develop an effective management system for tacit knowledge acquisition, sharing, and application, so as to improve the knowledge reserve ability of enterprises.

Further, in the case of strong task interdependence, the positive relationship between employees’ psychological capital and breakthrough innovation is more obvious. Our study found that when employees need information from others to complete their own work, their teams will communicate frequently, and they are more willing to share tacit knowledge. Because a success cannot rely on the efforts of one person, the good communication and cooperation of the team can effectively solve the difficulties encountered in the process of trying, especially when they cannot move forward. Therefore, knowledge hiding should be reduced and knowledge sharing should be increased by strengthening the task interdependence of the team.

## 6. Limitations and future research

Our study has several limitations that should be noted. First, the data collection of this study focused only on the Yunnan coffee industry, and whether the conclusion is generalizable remains to be verified. Future research can be explored from different industries, provinces or countries to expand the research scope. Second, in the selection of mediator variables, in addition to tacit knowledge sharing, the aspects of the acquisition, transfer, and the externalization of tacit knowledge can also be considered for future research. Moreover, future research may also select different moderator variables from different levels to further explore the influence mechanism between psychology capital and enterprise innovation. Finally, the dimensional division of the variables and the design of the questionnaire items will change according to the different scholars, and then produce different analysis results. Future research could select different scales to redesign the questionnaire and choose different samples to collect the data for analysis.

## Data availability statement

The raw data supporting the conclusions of this article will be made available by the authors, without undue reservation.

## Author contributions

RH contributed to the conception and design of the current study. JH performed the data collection. YL was responsible for drafting the manuscript, as well as the analysis and interpretation of data. YZ collected, analyzed the data, as well as helped to perform the analysis with constructive discussions. ED helped to revise and refine the manuscript. All authors contributed to the article and approved the submitted version.

## Funding

This study was supported by the National Natural Science Foundation of China (Award Nos.: 72271214 and 71762033) and the Yunnan Philosophy Social Science Foundation (YNPOPSS Award No.: ZD202213).

## Conflict of interest

The authors declare that the research was conducted in the absence of any commercial or financial relationships that could be construed as a potential conflict of interest.

## Publisher’s note

All claims expressed in this article are solely those of the authors and do not necessarily represent those of their affiliated organizations, or those of the publisher, the editors and the reviewers. Any product that may be evaluated in this article, or claim that may be made by its manufacturer, is not guaranteed or endorsed by the publisher.
